# Comparative Analysis of the Productivity and Immunogenicity of an Attenuated Classical Swine Fever Vaccine (LOM) and an Attenuated Live Marker Classical Swine Fever Vaccine (Flc-LOM-BE^rns^) from Laboratory to Pig Farm

**DOI:** 10.3390/vaccines9040381

**Published:** 2021-04-13

**Authors:** SeEun Choe, Ki-Sun Kim, Jihye Shin, Sok Song, Gyu-Nam Park, Ra Mi Cha, Sung-Hyun Choi, Byung-Il Jung, Kyung-Won Lee, Bang-Hun Hyun, Bong-Kyun Park, Dong-Jun An

**Affiliations:** 1 Virus Disease Division, Animal and Plant Quarantine Agency, Gimchen, Gyeongbuk-do 39660, Korea; ivvi59@korea.kr (S.C.); kisunkim@korea.kr (K.-S.K.); shinjibong227@gmail.com (J.S.); ssoboro@naver.com (S.S.); changep0418@gmail.com (G.-N.P.); rami.cha01@korea.kr (R.M.C.); hyunbh@korea.kr (B.-H.H.); parkx026@korea.kr (B.-K.P.); 2 Korea Pork Producers Association, Seocho-gu, Seoul 06643, Korea; heechan9@hanmail.net (S.-H.C.); exksa001@daum.net (B.-I.J.); 3 Pig Integrated Control Center (PICC), Cheonan City 31008, Korea; dukelee72@hanmail.net; 4 College of Veterinary Medicine, Seoul University, Gwanak-ro, Gwanak-gu, Seoul 08826, Korea

**Keywords:** CSFV, LOM, Flc-LOM-BE^rns^, SE, DIVA, pig

## Abstract

Herein, we compared the productivity of pigs inoculated with one of two classical swine fever (CSF) vaccines (low virulent of Miyagi (LOM) or Flc-LOM-BE^rns^) plus the swine *erysipelothrix rhusiopathiae* (SE) vaccine. The feed intake and weight increase of the pigs inoculated with Flc-LOM-BE^rns^ + SE were normal. However, the feed intake of the pigs inoculated with LOM + SE dropped sharply from four days post-vaccination (dpv). In addition, the slaughter date was an average of eight days later than that of the pigs inoculated with Flc-LOM-BE^rns^ + SE. All pigs inoculated with the Flc-LOM-BE^rns^ + SE vaccine were completely differentiated at 14 days against CSF E^rns^ antibody and at approximately 45 days against the bovine viral diarrhea virus (BVDV) E^rns^ antibody; the titers were maintained until slaughter. Leucopenia occurred temporarily in the LOM + SE group, but not in the Flc-LOM-BE^rns^ + SE group. Expression of tumor necrosis factor (TNF)-α and IFN-γ was significantly (*p* < 0.05) higher in the LOM + SE group than in the mock (no vaccine) group. When conducting the same experiment on a breeding farm, the results were similar to those of the laboratory experiments. In conclusion, the biggest advantage of replacing the CSF LOM vaccine with the Flc-LOM-BE^rns^ vaccine is improved productivity.

## 1. Introduction

Classical swine fever virus (CSFV; genus, *Pestivirus*; family, *Flaviviridae*) harbors a single-stranded, positive-sense RNA genome of approximately 12,300 nucleotides. CSFV is a highly contagious and fatal multi-systemic hemorrhagic disease of swine (*Sus scrofa*); it affects both breeding pigs and wild boars [1]. Differentiating infected from vaccinated animals (DIVA) vaccines, which are capable of triggering an immune response that is different from that triggered by the virulence strains, are of great interest [2]. In South Korea, all pigs must be inoculated with a classical swine fever (CSF) vaccine comprising a mixed live attenuated CSF (low virulent of Miyagi (LOM) strain) and SE (swine erysipelas, *Erysipelothrix rhusiopathiae*). The South Korean government has developed an attenuated live marker CSF vaccine (Flc-LOM-BE^rns^) that can be differentiated from virulent viruses to control CSF, and started to use it as a policy in 2020. A CSF live marker vaccine, Flc-LOM-BE^rns^, which functions as a DIVA, was developed by inserting the E^rns^ gene of bovine viral diarrhea virus (BVDV) into an infectious clone (Flc-LOM) based on the LOM (low virulent of Miyagi) strain used as a CSF vaccine in Korea [3]. A study in which early, middle, and late pregnancy sows were challenged with virulent CSFV after inoculation with the Flc-LOM-BE^rns^ vaccine showed that they were protected against vertical transmission of the viral infection to the fetus [3]. Lactogenic transfer of maternally derived antibodies from sows immunized with the Flc-LOM-BE^rns^ vaccine provided piglets with passive protection from virulent CSFV until they acquired active immunity [4]. Currently, all pigs in mainland South Korea (excluding Jeju island) receive either a mixed LOM plus swine *erysipelothrix rhusiopathiae* (SE) vaccine (LOM + SE) or a mixed Flc-LOM-BE^rns^ plus SE vaccine (Flc-LOM-BE^rns^ + SE) [4,5]. In the field, a single shot injection of a mixed Flc-LOM-BE^rns^ + SE vaccine is gradually being replaced the mixed LOM + SE vaccine [5]. However, the two vaccines (LOM + SE and Flc-LOM-BE^rns^ + SE) have not been evaluated with respect to seroconversion, persistence of neutralizing antibodies, time to formation of different antibodies, or body weight gain from the growing to the finishing stage. Although many studies have reported changes in body weight after inoculation of live vaccines such as porcine circovirus 2 (PCV2) [6] and *Lawsonina intracellularis* [7,8], few comparative reports have examined changes in body weight gain, cytokine expression, and vaccine safety in pigs receiving live attenuated virus vaccines. A previous study reported the induction of IFN-γ-producing T cells in vaccinated animals after CSF vaccination [9], whereas another showed that the expression of interleukin (IL)-10, tumor necrosis factor (TNF)-α, and IL-6 is related to CSFV pathogenesis [10]. 

The aim of this study was to investigate the possible use of the Flc-LOM-BE^rns^ vaccine as an emergency vaccine that triggers a sustained immune response and has DIVA functions. We also compared the food intake, body weight, body temperature changes, leucocyte counts, cytokine expression, and age at slaughter of pigs receiving either LOM + SE or Flc-LOM-BE^rns^ + SE in the Animal and Plant Quarantine Agency (APQA) laboratory and then re-tested in a commercial farm. 

## 2. Materials and Methods

### 2.1. CSF and SE Vaccines

This study used the commercial LOM + SE vaccine and the Flc-LOM-BE^rns^ + SE vaccine (the SuiShot^®^ LOM + SE-Live vaccine and SuiShot^®^ CSF Marker + SE-Live vaccine products from Choong Ang Vaccine Laboratories (CAVAC. Co., Daejeon, Korea), respectively). The SuiShot^®^ LOM + SE-Live vaccine consisted of CSF (LOM strain, >1 × 10^3^ TCID_50_ (Tissue Culture Infective Dose 50%)/mL) and SE (NL strain, >4 × 10^8^ CFU (Colony-Forming Unit)) in one dose (1 mL). The SuiShot^®^ CSF Marker + SE-Live vaccine contained CSF (Flc-LOM-BE^rns^ strain, >1 × 10^3.5^ TCID_50_/mL) and SE (NL strain, >4 × 10^8^ CFU) in one dose (1 mL).

### 2.2. Vaccination of Pigs with the Flc-LOM-BE^rns^ + SE and LOM + SE Vaccines at the APQA Laboratory

Pigs were divided into two vaccine groups (LOM + SE (*n* = 10) and Flc-LOM-BE^rns^ + SE (*n* = 10)) and monitored from vaccination until slaughter day at the APQA. Pigs were inoculated via the intramuscular route with one dose (1 mL) at 68 days old. Daily feed intake (grams) by all pigs within one room was measured using an automatic feed intake machine (Yeonhap Livestock Co. Ltd., Gimhae, South Korea). Feed intake (grams) per pig was calculated by dividing the total feed intake by the total number of pigs within one room. Feed intake capacity per day per pig was measured from before vaccination to the day of slaughter. To calculate the serum neutralizing antibody, CSFV E^rns^, and BVDV E^rns^ antibody titers, serum was collected at 0, 4, 14, 21, 30, 45, 74, and 109 dpv (days post-vaccination). To measure cytokine expression, a mock (no vaccine) pig group (68 days old, *n* = 10) was added for comparison with the two vaccination groups (LOM + SE and Flc-LOM-BE^rns^ + SE) for 7 days after vaccine. The expression of mRNA-encoding cytokines (IFN-γ, IL-10, TNF-α, and IL-6) was measured by real-time PCR (qRT-PCR) of blood samples collected at 7 dpv. The two vaccinated groups and the mock (no vaccine) group were also checked daily (from 0–10 dpv) for body temperature and leukocyte counts.

#### 2.2.1. Neutralization Tests and CSFV E2 c-ELISA 

The neutralization peroxidase linked assay (NPLA) was used to detect CSF-specific neutralizing antibodies in accordance with the standards manual of the World Organization for Animal Health [11]. The monoclonal antibody 3B6 (Median Diagnostics, Chuncheon, South Korea) was used to detect the expression of the CSF E2 protein by PK-15 cells. Neutralizing antibodies were determined to be at least 10-fold positive antibodies and less than 10-fold antibody negative according to the OIE (World Organization for Animal Health) criteria. The VDPro^®^ CSFV AB C-ELISA (MEDIAN Diagnostic Co. Cat No. ES-CSF-02, Chuncheon, Korea), a competitive (c)-ELISA designed to detect the E2 protein, was also used as an indicator of the PC value (≥40% positive and <40% negative). 

#### 2.2.2. CSFV E^rns^ ELISA and BVDV E^rns^ ELISAs 

The CSFV E^rns^ and BVDV BE^rns^ ELISAs used to detect specific antibodies were performed as described previously [3,4]. The VDPro^®^ CSF E^rns^ Ab b-ELISA (MEDIAN Diagnostic Co., Cat No. ES-CSF-O5, Chuncheon, Korea), a c-ELISA designed to detect the E^rns^ protein, was also used to determine the S/N (sample O.D/negative control O.D) value (>0.5 negative and ≤0.5 positive). The VDPro^®^ BVDV E^rns^ A bi-ELISA (MEDIAN Diagnostic Co., Cat No. ES-CSF-O5, Korea), which is based on the E2 protein, provided an S/P (sample O.D/positive control O.D) ratio (≥0.6 positive and <0.6 negative).

#### 2.2.3. Cytokine Expression in Pig Blood 

Expression of cytokine genes was examined in pigs inoculated with the two CSF vaccines (Flc-LOM-BE^rns^ + SE and LOM + SE) and in the mock pigs. Peripheral blood mononuclear cells (PBMCs) were collected on different dpv and the expression of IFN-γ, IL-6, IL-10, and TNF-α mRNA was measured by qRT-PCR, as described previously [12,13] (Table 1). 

Briefly, 10 mL of PBS (phosphate-buffered saline) was mixed with 10 mL of collected blood and then overlayered with 4 mL of lympho-prep. After centrifugation for 20 min at 1800 rpm at room temperature, the leukocyte layer (buffy coat) was placed into a new tube and washed with PBS. Total RNA was extracted from the PBMCs using a Qiagen RNeasy mini prep kit. The concentration of the extracted RNA was measured in a spectrophotometer and cDNA was synthesized using a Nanohelix cDNA synthesis kit. qRT-PCR was performed by mixing 1 mL of synthesized cDNA with 500 nM of forward and reverse primers and 1 × SSO Advanced Universal Probes Supermix (Bio-Rad, Hercules, CA, USA, 172-5280) [12,13] (Table 1). The β-actin and GAPDH were used as internal controls [13] (Table 1). Relative quantification (RQ) of mRNA expression was calculated by the 2^−ΔΔCt^ method [14].

### 2.3. Vaccination of Pigs on a Pig Farm with Flc-LOM-BE^rns^ + SE and LOM + SE

Eighty pigs on the Pungil (PI) farm located in Cheonan city were inoculated (*n* = 40 pigs per group; age, 81 days) via the intramuscular route with one dose (1 mL) and feed intake, body weight, leucocyte count, and age at slaughter were monitored. Pig blood samples were collected at 0, 30, 90, and 120 dpv to evaluate serum neutralizing antibodies and CSFV E^rns^ and BVDV E^rns^ titers.

### 2.4. Statistical Analysis

All statistical analyses were performed using the GraphPad Prism software, version 6.0, for Windows. Data are expressed as the mean ± standard error (SEM) and significant differences (*p* < 0.05 and *p* < 0.01) are indicated by one or two asterisks.

## 3. Results

### 3.1. Changes in Body Weight and Feed Intake after Administration of the Two CSF Vaccines at the APQA Laboratory

**Figure 1:**
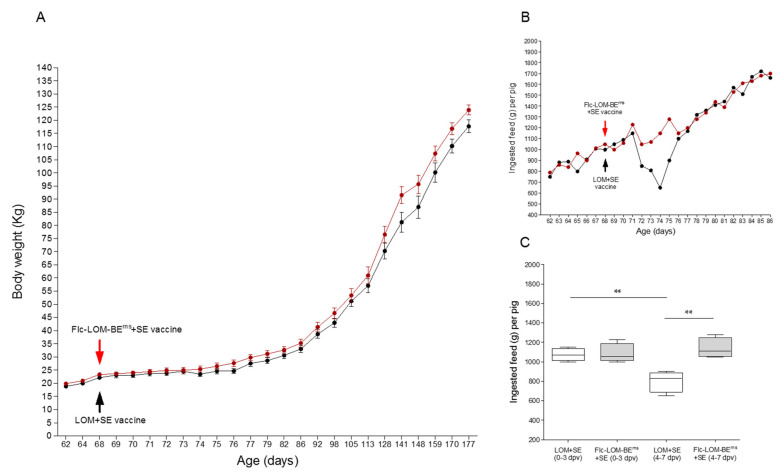
Comparison of the weight and food intake between pigs vaccinated with low virulent of Miyagi (LOM) + swine *erysipelothrix rhusiopathiae* (SE) or Flc-LOM-BE^rns^ + SE at the Animal and Plant Quarantine Agency (APQA) laboratory. Body weight and age (**A**), food intake per day (**B**), and intake rate (**C**). The LOM + SE and Flc-LOM-BE^rns^ + SE vaccines are shown in black and red, respectively. ** *p* < 0.01.

Prior to vaccination, the average weight of the 68-day-old pigs in the Flc-LOM-BE^rns^ + SE group was 18.83 ± 0.91 kg, whereas that in the LOM + SE group was 19.85 ± 1.59 kg (Figure 1A). 

**Figure 2:**
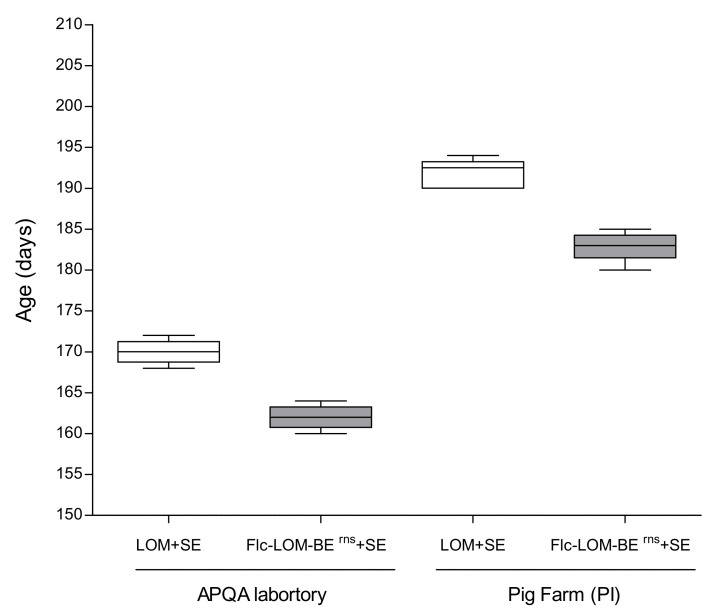
Comparison of slaughter age (days) between pigs vaccinated with LOM + SE or Flc-LOM-BE^rns^ + SE at the APQA laboratory and pig farm (Pungil (PI)). Data represent the mean ± standard error.

The average weight after Flc-LOM-BE^rns^ + SE vaccination was 110.2 ± 2.712 kg and pigs were slaughtered at 162 days old; however, pigs in the LOM + SE vaccine group reached 110.6 ± 2.958 kg at 170 days old (eight days later) (Figure 1A and Figure 2). The average daily weight gain (ADG) of the pigs inoculated with the LOM + SE vaccine was 0.484 kg (0–5 dpv), 0.812 kg (6–45 dpv), and 0.932 kg (46–102 dpv), whereas the ADG of pigs inoculated with Flc-LOM-BE^rns^ + SE was 0.326 kg (0–5 dpv), 0.903 kg (6–45 dpv), and 0.978 kg (46–102 dpv). Initially, there was no difference in feed intake between the groups (daily average = 1085 ± 50.1 g/pig in the Flc-LOM-BE^rns^ + SE group and 1073 ± 31.7 g/pig in the LOM + SE group, from 0 to 3 dpv) (Figure 1B,C). However, there was a difference between 4 and 7 dpv; the average daily feed intake of pigs inoculated with Flc-LOM-BE^rns^ + SE was 1138 ± 48.7 g/pig, whereas that of pigs inoculated with the LOM + SE vaccine was 802 ± 54.1 g/pig (Figure 1B,C).

### 3.2. Positive Seroconversion and Differences in E^rns^ Antibody Production 

**Figure 3:**
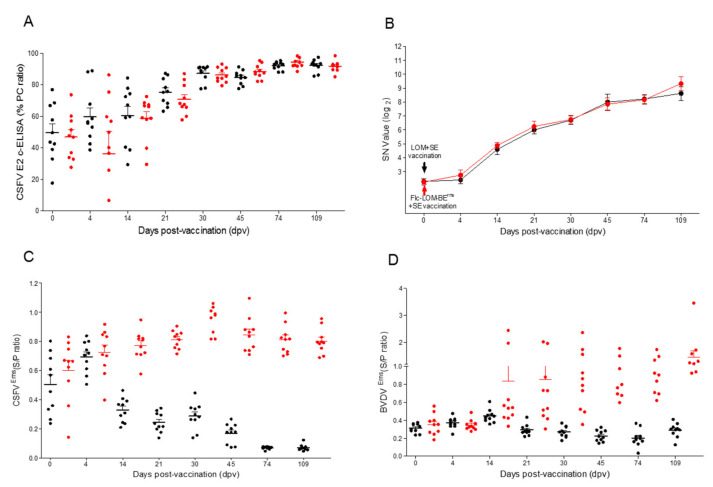
Antibodies induced by the LOM + SE and Flc-LOM-BE^rns^ + SE vaccines at the APQA laboratory. Classical swine fever (CSF) E2 antibodies (**A**), serum neutralizing antibodies (**B**), CSF E^rns^ antibodies (**C**), and bovine viral diarrhea (BVD) E^rns^ antibodies (**D**). The LOM + SE and Flc-LOM-BE^rns^ + SE vaccines are shown in black and red dots, respectively. CSFV, classical swine fever virus. PC (positive control) and S/P ratio are positive control and sample O.D/positive control O.D, respectively.

Before vaccination, the pigs had low maternal transfer CSF antibodies, which were confirmed by cELISA, NPLA, and CSF E^rns^ ELISA (Figure 3A–C). All pigs inoculated with the LOM + SE or Flc-LOM-BE^rns^ + SE vaccine were positive for E2 antibodies at 21 dpv using the CSF c-ELISA kit (Figure 3A). However, an NPLA assay revealed that all pigs in both groups were positive for neutralizing antibodies (≥4 log2) at 14 dpv (Figure 3B). The LOM + SE-vaccinated pigs were positive for CSF E^rns^ antibodies at 14 dpv, whereas the Flc-LOM-BE^rns^ + SE-vaccinated pigs were negative (Figure 3C). Only 20% of the pigs in the Flc-LOM-BE^rns^ + SE group had bovine viral diarrhea (BVD) E^rns^ antibodies at 14 dpv; however, all animals in this group seroconverted at 45 dpv (Figure 3D). The persistence of neutralizing antibodies in the serum remained high (titer ≥8 log2) until the day of slaughter, as did the titers of BVD E^rns^ (Figure 3).

### 3.3. Changes in Body Temperature and Leukocyte Counts

**Figure 4:**
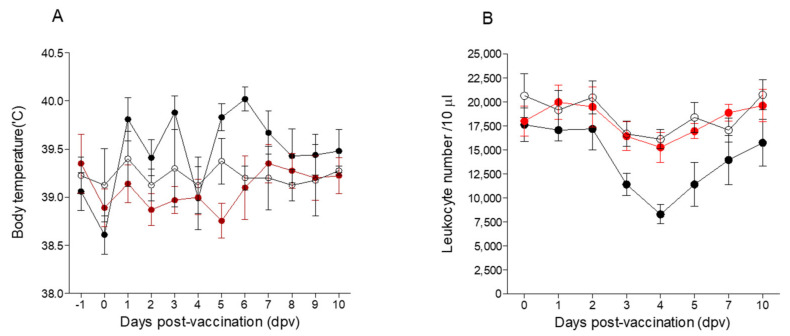
Changes in the body temperature and leukocyte counts post-vaccination. Body temperature (**A**) and leukocyte counts (**B**). The LOM + SE and Flc-LOM-BE^rns^ + SE vaccines are shown in light gray and white bars, respectively (**A**). The LOM + SE, Flc-LOM-BE^rns^ + SE, and mock vaccines (no vaccine) are shown in black, red, and white dots, respectively (**B**). Data represent the mean ± standard error.

The average body temperature of the pigs inoculated with the Flc-LOM-BE^rns^ + SE vaccine was 38.3–39.8 °C across the 10-day observation period post-vaccination (Figure 4A). That of the LOM + SE vaccine group was slightly higher (38.1–40.3 °C) (Figure 4A). The leukocyte counts of the pigs inoculated with the Flc-LOM-BE^rns^ + SE- or mock-vaccinated (no vaccine) pigs were 15,000–21,200/10 μL, with no changes noted up until 10 dpv (Figure 4B). However, the leukocyte counts in the pigs inoculated with the LOM + SE vaccine began to drop from 2 dpv; the count fell to 8000/10 μL on 4 dpv, before returning to normal from 5 dpv (Figure 4B).

### 3.4. Changes in Cytokine Gene Expression 

**Figure 5:**
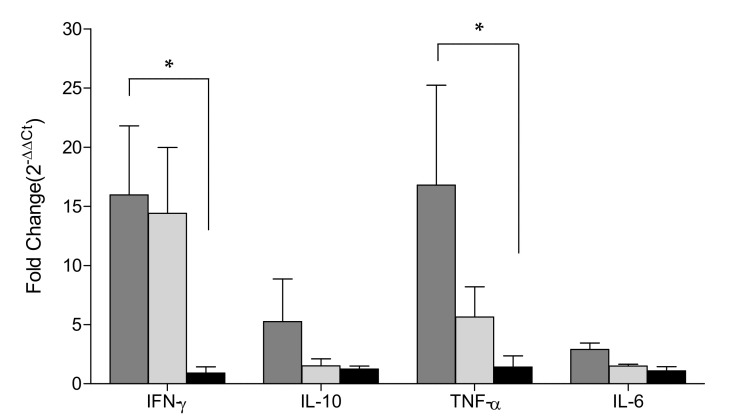
Expression of cytokines (IFN-γ, IL-10, TNF-α, and IL-6) at seven days post-vaccination. Relative mRNA expression was calculated using the 2^−ΔΔCt^ method. The LOM + SE, Flc-LOM-BE^rns^ + SE, and mock groups are denoted by gray, light gray, and black bars, respectively. * *p* < 0.05.

The relative quantification (RQ) (2^−ΔΔCt^) of interferon-γ (IFN-γ) mRNA expression was 11.14 ± 5.57 for the pigs in the Flc-LOM-BE^rns^ + SE group and 11.65 ± 5.83 for the pigs in the LOM + SE group; the differences were significant (*p* < 0.05) when compared to the expression in the mock (no vaccine) pigs (1.03 ± 0.51) (Figure 5). 

However, at 7 dpv, the 2^−ΔΔCt^ of interleukin-10 (IL-10) and interleukin-6 (IL-6) mRNA expression was somewhat higher in the LOM + SE group (7.22 ± 3.61 for IL-10 and 1.07 ± 0.54 for IL-6) than in the Flc-LOM-BE^rns^ + SE group (1.19 ± 0.60 for IL-10 and 0.29 ± 0.15 for IL-6) (Figure 5). On 7 dpv, the expression of tumor necrosis factor-α (TNF-α) in the pigs inoculated with LOM + SE (16.86 ± 8.43) was significantly higher than that in the pigs inoculated with Flc-LOM-BE^rns^ + SE (5.12 ± 2.56); the difference between the LOM + SE and mock (no vaccine) pigs was significant (*p* < 0.05) (Figure 5). 

### 3.5. Reproducibility of the Results on a Breeding Pig Farm

**Figure 6:**
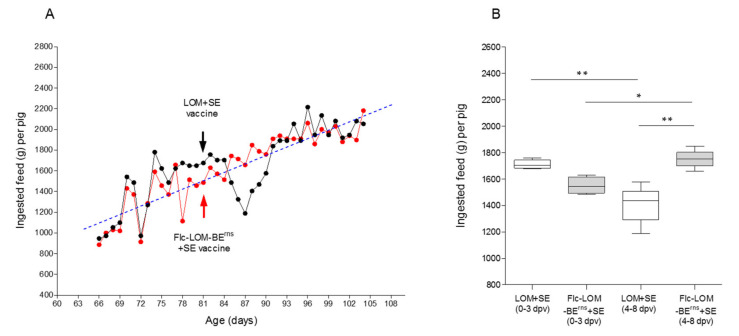
Food intake by pigs vaccinated with the LOM + SE and Flc-LOM-BE^rns^ + SE vaccines on a pig farm. Food intake per day (**A**) and intake rate (**B**). The LOM + SE and Flc-LOM-BE^rns^ + SE vaccines are denoted by black and red, respectively. * *p* < 0.05 and ** *p* < 0.01.

Both vaccines (Flc-LOM-BE^rns^ + SE and LOM + SE) were used to inoculate 81-day-old pigs on a pig farm; the number of pigs inoculated with each vaccine was 40 (Figure 6A). 

**Figure 7:**
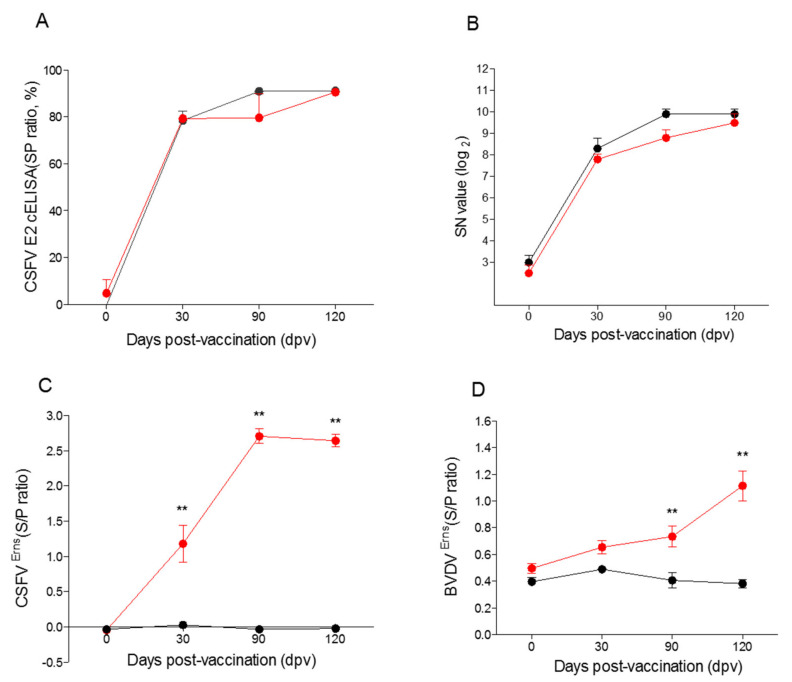
Antibodies induced by the two CSF vaccines used on the pig farm. CSF E2 antibodies (**A**), serum neutralizing antibodies (**B**), CSF E^rns^ antibodies (**C**), and BVD E^rns^ antibodies (**D**). The LOM + SE and Flc-LOM-BE^rns^ + SE vaccines are marked in black and red, respectively. Data represent the mean ± standard error and ** indicates significant differences (*p* < 0.001).

Before vaccination, the pigs had no maternal transfer CSF antibodies (Figure 7A–C). There was no change in feed intake at 0–3 dpv in the LOM + SE group (1710 ± 16.9 g/pig); however, the feed intake at 4–8 dpv fell sharply (1408 ± 55.7 g/pig) (Figure 6B). In contrast, the Flc-LOM-BE^rns^ + SE vaccine group showed increased feed intake (1751 ± 26.5 g/pig) at 4–8 dpv compared to 0–3 dpv (1550 ± 16.9 g/pig) (Figure 6B). On average, the pigs in the LOM + SE vaccination group were slaughtered at 192 days old (110.4 ± 3.354 kg), whereas those in the Flc-LOM-BE^rns^ + SE vaccination group were slaughtered at 183 days old (110.5 ± 3.049 kg) (Figure 2).

The E2 antibody S/P ratio in the CSF c-ELISA was positive for both vaccines (average >80%) from 30 dpv (Figure 7A). The mean serum neutralizing antibody titer in the pigs (aged 80 days) inoculated with the Flc-LOM-BE^rns^ + SE vaccine was <4 (log 2) at 0 dpv, 7.33 (log 2) at 30 dpv, 6.33 (log 2) at 90 dpv, and 5.5 (log 2) at 120 dpv (Figure 7B). At 30 dpv, the S/P ratio for the different diagnostic antibodies was as follows: 0.9–1.5 for CSF E^rns^ antibodies in the pigs inoculated with LOM + SE, and 0.6–0.7 for BVD E^rns^ antibodies in the pigs inoculated with Flc-LOM-BE^rns^ + SE (Figure 7C,D).

## 4. Discussion

The vaccination of domestic pigs with a CSF vaccine (LOM strain) has been implemented nationally by the South Korean government since 1976; this vaccine shows good immunogenicity, but safety-related problems continue to arise [15,16,17,18]. In South Korea, a CSF antibody survey after inoculation with the LOM vaccine is carried out at least two or three times per year on all pig farms and slaughterhouses [19]. The CSF antibody positivity rate is >95% nationwide, thereby suppressing virulent CSFV [19]. Herein, we found that the CSF-neutralizing antibody titer in pigs inoculated with the Flc-LOM-BE^rns^ + SE vaccine at the APQA was 4.8 ± 0.6 (log_2_) on 14 dpv and 9.3 ± 1.2 (log_2_) on 109 dpv (177 days old), whereas that of pigs on the pig farm was 9.4 ± 0.3 (log_2_) on 120 dpv (201 days old). This suggests that the immunogenicity and persistence of the Flc-LOM-BE^rns^ and LOM vaccines are very similar. 

Currently, the live marker vaccine (CP7_E2alf) is used in Europe to differentiate CSF vaccines with DIVA function [20,21]. The onset of detectable E2-specific neutralizing antibodies in CP7_E2alf-vaccinated pigs is detectable from 8 dpv, and the first E2 antibodies are detected by an E2 c-ELISA from 11 dpv [21]. Usually, “CP7_E2alf” induces antibodies within the first two weeks post-vaccination; these antibodies persist for at least six months [20]. When we inoculated CSF antibody-negative pigs with the Flc-LOM-BE^rns^ vaccine, no CSF E^rns^ antibodies appeared; however, seroconversion to BVD E^rns^ antibodies appeared partially at 14 dpv and was complete by 45 dpv. Since these differential antibodies (CSF E^rns^ and BVD E^rns^) are maintained until slaughter, we suggest that the Flc-LOM-BE^rns^ vaccine functions adequately as a DIVA vaccine. 

Up until 10 dpv, there were no changes in body temperature or the number of leukocytes (38.3–39.8 °C and 15,000–21,200/10 μL, respectively) in response to the Flc-LOM-BE^rns^ + SE vaccine; however, there were temporary leukocyte changes (8000/10 μL) in response to the LOM + SE vaccine. Other studies have reported a transient leucopenia after administration of the LOM vaccine [15,16,17,18]. 

TNF-α and IL-6 are thought to contribute to hemorrhage, a characteristic of acute CSF disease [22]. Previous studies have shown that the C-strain vaccine induces IFN-γ production post-vaccination [23,24], which serves as a good marker for anti-CSFV cell-mediated responses. During CSFV infection, IL-10 facilitates immunosuppression and viral persistence by inhibiting Th1 and NK cells [25,26]. Unvaccinated pigs infected with CSFV have significantly higher TNF-α, IL-10, and IL-6 levels (*p* = 0.001) than vaccinated pigs [27]. However, the expression of IFN-γ in vaccinated pigs is significantly higher (*p* = 0.001) than that in unvaccinated pigs infected with CSFV [27]. Herein, we found that the expression of IL-6, IL-10, and TNF-α at 7 dpv was slightly higher in the pigs receiving the LOM + SE vaccine. However, lower expression of IL-6, IL-10, and TNF-α in the pigs inoculated with the Flc-LOM-BE^rns^ + SE vaccine may indicate reduced inflammatory activation and reduced protection against virulent CSFV. The similar expression level of IFN-γ between the two groups may indicate a strong immune response to both vaccines, unless the actual level of IFN-γ is different to the mRNA expression level. Although the LOM vaccine has an excellent immunogenic effect, studies have suggested that giving it to 55–70-day-old pigs infected with respiratory disease (e.g., PCV2 (porcine circovirus 2), PRRS (porcine reproductive and respiratory syndrome), and mycoplasma) would worsen respiratory symptoms due to the immunosuppressive effects [17,18]. 

From 4 to 8–9 dpv, the feed intake by the LOM + SE-vaccinated group fell sharply and activity slowed down; the pigs reached slaughter weight (110 kg) eventually, but this was delayed, causing economic losses. However, the Flc-LOM-BE^rns^ + SE pigs fed normally and reached slaughter weight eight to nine days earlier than the LOM + SE pigs. The monetary value of the difference between both groups at the time to reach slaughter (around eight days) was estimated to be approximately $6.25 (U.S.) when converting only the feed cost reduction per pig. Up to five days after administration of the two vaccines, the ADG of the LOM + SE pigs was 0.158 kg/day more than that of Flc-LOM-BE^rns^ + SE pigs, but the ADG of Flc-LOM-BE^rns^ + SE pigs was 0.091 kg/day more during the growing period (6–45 dpv; 74–113 days old) and 0.046 kg/day more during the finishing period (46–102 dpv; 114–170 days old) than the ADG of the LOM + SE pigs. A previous study reported that the PCV2 vaccination had a significant effect on the ADG of finishing pigs (41.5 g) and nursery finishing pigs (33.6 g), with only a 10.6 g increase noted for nursery pigs [6]. 

The Korean government mandates the use of CSF vaccines on pig farms, and a fine is imposed if animals are under 80% antibody-positive. This policy of compulsory CSF vaccination can affect the rate of weight increase, which can delay the day of slaughter. Therefore, it is essential to replace the CSF vaccine (LOM strain) with a new live marker CSF vaccine (i.e., the Flc-LOM-BE^rns^ strain) to improve weight gain and safety. 

## 5. Conclusions

The Flc-LOM-BE^rns^ vaccine allows pigs to reach slaughter weight eight to nine days earlier than the existing LOM vaccine strain, which reduces pig feed costs. Therefore, the Flc-LOM-BE^rns^ vaccine has advantages not only in terms of DIVA function, but also provides excellent passive immunity and persistent active immunity; this makes it useful as an emergency vaccine with economic benefits for pig farmers.

## Figures and Tables

**Table 1:** Primers and probes used for real-time PCR to detect porcine cytokines.

Gene	Primer (5′–3′)	Probe (5′FAM and 3′BHQ-1)	Size (bp)	Reference
IFN-γ	F: CGATCCTAAAGGACTATTTTAATGCAA R: TTTTGTCACTCTCCTCTTTCCAAT	ACCTCAGATGTACCTAATGGTGGACCTCTT	102	[12]
IL-6	F: CTGGCAGAAAACAACCTGAACC R: TGATTCTCATCAAGCAGGTCTCC	TGGCAGAAAAAGACGGATGC	93	[13]
IL-10	F: CGGCGCTGTCATCAATTTCTG R: CCCCTCTCTTGGAGCTTGCTA	AGGCACTCTTCACCTCCTCCACGGC	89	[12]
TNF-α	F: AACCTCAGATAAGCCCGTCG R: ACCACCAGCTGGTTGTCTTT	CCAATGCCCTCCTGGCCAAC	128	[13]
β-Actin (control)	F: AGCGCAAGTACTCCGTGTG R: CGGACTCATCGTACTCCTGCTT	TCGCTGTCCACCTTCCAGCAGATGT	105	[13]
GAPDH (control)	F: ACATGGCCTCCAAGGAGTAAGA R: GATCGAGTTGGGGCTGTGACT	CCACCAACCCCAGCAAGAGCACGC	105	[13]

## Data Availability

The data presented in this study are available on request from the corresponding author.
